# Nasal carriage of CTX-M-55-producing *Escherichia coli* ST8369 in a healthy cohort in the city of Yangzhou, China

**DOI:** 10.3389/fcimb.2022.970940

**Published:** 2022-08-03

**Authors:** Zhen-Yu Wang, Yue Jiang, Yi-Qiao Shao, Heng-Fan Lu, Meng-Jun Lu, Xinan Jiao, Qiu-Chun Li, Jing Wang

**Affiliations:** ^1^ Jiangsu Key Laboratory of Zoonosis, Jiangsu Co-Innovation Center for Prevention and Control of Important Animal Infectious Diseases and Zoonoses, Yangzhou University, Yangzhou, China; ^2^ Key Laboratory of Prevention and Control of Biological Hazard Factors (Animal Origin) for Agrifood Safety and Quality, Ministry of Agriculture of China, Yangzhou University, Yangzhou, China

**Keywords:** *bla*_CTX-M_, chromosome, *Escherichia coli*, IS*Ecp1*, plasmids, ST8369

## Abstract

This study aimed to investigate the prevalence and diversity of extended-spectrum β-lactamases (ESBL)-producing *Escherichia coli* isolates from healthy individuals in a community and to elucidate their dissemination mechanism. Cefotaxime-resistant *E. coli* were isolated from 95 samples of healthy persons from one community in Yangzhou, China, and were tested for minimal inhibitory concentrations of 14 antimicrobial agents. The isolates were subjected to whole genome sequencing by Illumina Hiseq or PacBio single-molecule real-time sequencing. A total of 30 cefotaxime-resistant *E. coli* isolates were obtained, carrying *bla*_CTX-M_ (n=29) or *bla*_DHA_ (n=1), of which the *bla*_CTX-M-55_ (n=19) was the most predominant genotype. One novel *bla*_CTX-M_ variant *bla*_CTX-M-252_ was identified. Thirteen CTX-M-55-producing *E. coli* isolates belonged to ST8369 from nasal (n=12) or faecal (n=1) samples shared the identical cgMLST type, resistance profiles, resistance genes, plasmid replicons, and a 5,053-bp *bla*_CTX-M-55_ structure ΔIS*26*-ΔIS*Ecp1*-*bla*_CTX-M-55_-Δ*orf477*-ΔTn*2*. The *bla*_CTX-M-55_ gene was located on IncHI2/ST3 plasmid in *E. coli* ST8369. The lengths of *bla*_CTX-M_/*bla*_DHA_-carrying contigs in the remaining 17 *E. coli* strains ranged from 1,663 to 382,836 bp, located on chromosome (n=4) or plasmids (n=5); the location of the other eight contigs could not be determined due to incomplete assembly. The *bla*_CTX-M_ was associated with IS*Ecp1* as previously reported. Nasal colonization of CTX-M-55-producing ST8369 *E. coli* strains has occurred among healthy individuals in one community. There is a potential risk of antimicrobial resistance dissemination between humans within one community through close contact or environment *via* aerosols or dust. Therefore, surveillance of nasal carriage of *bla*_CTX-M_ in communities is warranted to further monitor the spread of the antimicrobial resistance genes in China.

## Introduction

Extended-spectrum cephalosporins are widely used in human clinics and veterinary medicine to treat infections caused by multidrug-resistant Gram-negative bacteria; thus, extended-spectrum β-lactamases (ESBL), particularly CTX-M enzymes, have been increasingly reported in human clinical settings and animals worldwide (Bevan et al., 2017). Globally, incidence of CTX-M ESBLs is increasing, *bla*_CTX-M-15_ and *bla*_CTX-M-14_ are the predominant genotypes detected in many parts of the world (Woerther et al., 2013; Bevan et al., 2017). The global dissemination of *bla*_CTX-M_ is mainly due to the rapid horizontal transfer mediated by conjugative plasmids; the epidemic plasmids such as IncF, IncI, and IncHI2 facilitate the global spread of *bla*_CTX-M_ in Enterobacteriaceae from humans, animals and the environment, particularly in *Escherichia coli* (Bevan et al., 2017; Rozwandowicz et al., 2018; Partridge et al., 2018). Mobile elements such as ISEcp1, IS26, and ISCR1 have also played an essential role in the blaCTX-M transmission (Bevan et al., 2017; Partridge et al., 2018). In addition, some successful *E. coli* clones, such as the *E. coli* clone ST131 lineage diffused worldwide, are also responsible for *bla*_CTX-M-15_ global dissemination, mostly in human clinics (Bevan et al., 2017).

To date, many studies have focused on CTX-M-producing Enterobacteriaceae from clinical patients. However, the high prevalence of CTX-M-producing *E. coli* colonizing the intestinal tract of healthy persons in communities is of particular concern, since they could be a major reservoir of *bla*_CTX-M_ (Woerther et al., 2013; Bevan et al., 2017; Chen et al., 2021). In this study, we aimed to investigate the prevalence and characterization of extended-spectrum β-lactamases (ESBL)-producing *E. coli* isolates from healthy individuals in a community from Yangzhou, China, to elucidate their dissemination mechanism within this small-scale community.

## Materials and methods

### Sample collection and antimicrobial susceptibility testing

From April 9^th^ to May 17^th^ 2021, 58 fecal samples and 37 nasal swabs of 72 healthy volunteers (3 male and 69 female) with no obvious disease symptoms at the age of 15-46 were collected from 37 apartments located in three buildings in one community in Yangzhou, China. Participants had been exposed to antimicrobial agents in the three months prior to sample collection were excluded from this study. This small-scale community with approximately 2000 individuals located in the urban area of Yangzhou, and included three main areas, and one building was randomly selected to sample in each area (**Supplementary Figure S1**). Individual written informed consent for samples was obtained from all volunteers. Samples were incubated in LB broth (OXOID, Basingstoke, UK) for 18~24 h and then cultured on the MacConkey agar (Haibo, Qingdao, China) with 2 mg/L cefotaxime. One *E. coli* isolate per plate was selected and identified by 16S rRNA gene sequencing using PCR and Sanger sequencing (Kim et al., 2010). The cefotaxime-resistant *E. coli* isolates were tested susceptibility to 14 antimicrobial agents including ampicillin, cefotaxime, meropenem, gentamicin, amikacin, streptomycin, tetracycline, chloramphenicol, florfenicol, nalidixic acid, ciprofloxacin, colistin, fosfomycin, and sulfamethoxazole/trimethoprim by using the agar dilution or broth microdilution method (limited to colistin). The results were interpreted according to Clinical Laboratory Standards Institute (CLSI) M100, 30^th^ edition (CLSI, 2020). *E. coli* ATCC 25922 was used as the quality control strain.

### Whole genome sequencing and analysis

All cefotaxime-resistant *E. coli* isolates were sequenced by Illumina Hiseq. The library was constructed using NEB NEXT Ultra DNA Library Prep Kit for Illumina (New England Biolabs, USA) and 150 bp paired-end reads were obtained. For each *E. coli* isolate performed WGS, at least 100-fold coverage of raw reads was collected. The 150 bp pair-end raw reads were trimmed and filtered by the NGSQC toolkit 2.3.3, then were assembled into contigs using SPAdes 3.8.2 (Bankevich et al., 2012). One representative ST8369 *E. coli* isolate YZ21HCE18 was sequenced using PacBio single-molecule real-time sequencing. The phylogenetic groups of *E. coli* were confirmed according to previously described protocol by using assembled contigs (Clermont et al., 2000). The genomes were subjected to analysis of multilocus sequence typing (MLST), core genome multilocus sequencing typing (cgMLST), resistance genes, mutations and plasmids by using the Center for Genomic Epidemiology (CGE) pipelines (http://www.genomicepidemiology/org/). The phylogenetic tree of these isolates was constructed using Parsnp (https://harvest.readthedocs.io/en/latest/content/parsnp.html) and visualized by iTOL (Letunic and Bork, 2016). The *bla*_CTX-M/_ *bla*_DHA_-carrying contigs were retrieved from the draft genomes and analyzed by ISfinder (https://www-is.biotoul.fr/) and BLAST (https://blast.ncbi.nlm.nih.gov/Blast.cgi). The *bla*_CTX-M_-bearing plasmid pYUYZ18-1 in strain YZ21HCE18 was compared with other ST8369 *E. coli* isolates using BRIG.

### Conjugation assay

The transferability of cefotaxime resistance was determined using conjugation experiments as previously described (Chen et al., 2007) and streptomycin-resistant *E. coli* C600 as the recipient. Transconjugants were selected using 2 mg/L cefotaxime and 3,000 mg/L streptomycin, and were confirmed by detecting *bla*_CTX-M_ or *bla*_DHA-1_ using PCR and sequencing (Chen et al., 2004; Liu et al., 2007).

## Results

### Prevalence and genotype distribution of cefotaxime-resistant *E. coli*

Thirty cefotaxime-resistant *E. coli* isolates were obtained from 12 nasal swabs and 18 fecal samples from 27 individuals (**Table 1**). The *bla*_CTX-M-55_ (n=19) gene was identified as the most predominant genotype, followed by *bla*_CTX-M-14_ (n=5), *bla*_CTX-M-65_ (n=2), *bla*_CTX-M-15_ (n=1), *bla*_CTX-M-64_ (n=1), and *bla*_DHA-1_ (n=1) (**Table 1**). One novel *bla*_CTX-M_ variant *bla*_CTX-M-252_ (GenBank accession no. OL884447) was identified, and differed from *bla*_CTX-M-65_ by a single nucleotide resulting in one amino acid change (A32V).

**Table 1:** Chracterization of cefotaxime-resistant *Escherichia coli* isolates in this study.

Strains^a^	Source^b^	Sampling Date	MLST	Phylogenetic group	ESBL genotype	Antimicrobial susceptibility profile^c^	Plasmid	Mutations^d^	Length of *bla*_CTX/DHA_-contig (bp)
YZ21HCE7*	nasal swab, F	2021/5/14	8369	B1	*bla*_CTX-M-55_	AMP/CTX/GEN/STR/TET/CHL/FFC/SXT	IncHI2, F102:A17:B-	N	5,053
YZ21HCE8*	nasal swab, F	2021/5/14	8369	B1	*bla*_CTX-M-55_	AMP/CTX/GEN/STR/TET/CHL/FFC/SXT	IncHI2, F102:A17:B-	N	5,053
YZ21HCE9*	nasal swab, F	2021/5/14	8369	B1	*bla*_CTX-M-55_	AMP/CTX/GEN/STR/TET/CHL/FFC/SXT	IncHI2, F102:A17:B-	N	5,053
YZ21HCE18*	nasal swab, F	2021/5/14	8369	B1	*bla*_CTX-M-55_	AMP/CTX/GEN/STR/TET/CHL/FFC/SXT	IncHI2, F102:A17:B-	N	5,053
YZ21HCE19*	nasal swab, F	2021/5/14	8369	B1	*bla*_CTX-M-55_	AMP/CTX/GEN/STR/TET/CHL/FFC/SXT	IncHI2, F102:A17:B-	N	5,053
YZ21HCE21*	nasal swab, F	2021/5/16	8369	B1	*bla*_CTX-M-55_	AMP/CTX/GEN/STR/TET/CHL/FFC/SXT	IncHI2, F102:A17:B-	N	5,053
YZ21HCE22*	nasal swab, F	2021/5/16	8369	B1	*bla*_CTX-M-55_	AMP/CTX/GEN/STR/TET/CHL/FFC/SXT	IncHI2, F102:A17:B-	N	5,053
YZ21HCE26*	feces, F	2021/5/16	8369	B1	*bla*_CTX-M-55_	AMP/CTX/GEN/STR/TET/CHL/FFC/SXT	IncHI2, F102:A17:B-	N	5,053
YZ21HCE27*	nasal swab, F	2021/5/17	8369	B1	*bla*_CTX-M-55_	AMP/CTX/GEN/STR/TET/CHL/FFC/SXT	IncHI2, F102:A17:B-	N	5,053
YZ21HCE28*	nasal swab, F	2021/5/17	8369	B1	*bla*_CTX-M-55_	AMP/CTX/GEN/STR/TET/CHL/FFC/SXT	IncHI2, F102:A17:B-	N	5,053
YZ21HCE29*	nasal swab,M	2021/5/17	8369	B1	*bla*_CTX-M-55_	AMP/CTX/GEN/STR/TET/CHL/FFC/SXT	IncHI2, F102:A17:B-	N	5,053
YZ21HCE30*	nasal swab,M	2021/5/17	8369	B1	*bla*_CTX-M-55_	AMP/CTX/GEN/STR/TET/CHL/FFC/SXT	IncHI2, F102:A17:B-	N	5,053
YZ21HCE31*	nasal swab, F	2021/5/17	8369	B1	*bla*_CTX-M-55_	AMP/CTX/GEN/STR/TET/CHL/FFC/SXT	IncHI2, F102:A17:B-	N	5,053
YZ21HCE1*	feces, F	2021/4/9	773	A	*bla*_CTX-M-14_	AMP/CTX/NAL/CIP/FOS	F2:A-:B10, IncX1, Col(BS512)	*gyrA*(S83L+D87N), *parC* (S80I), *parE* (S458A)	1,687
YZ21HCE2*	feces, F	2021/4/9	453	B1	*bla*_DHA-1_	AMP/CTX/STR/TET/NAL	IncHI2, IncFII, IncFIB	N	18,905
YZ21HCE3*	feces, F	2021/4/9	5614	B1	*bla*_CTX-M-15_	AMP/CTX/STR/SXT	IncK	N	35,054
YZ21HCE4	feces, F	2021/4/10	1434	A	*bla*_CTX-M-14_	AMP/CTX/NAL	IncY, IncX1, IncFIB(K), Col156, IncFII(pCRY)	N	21,609
YZ21HCE5	feces, F	2021/4/10	95	B2	*bla*_CTX-M-65_	AMP/CTX/GEN/STR/TET/CHL/FFC/NAL/SXT	IncHI2, F18:A-:B1:C4	*gyrA* (S83L)	3,019
YZ21HCE6*	feces, F	2021/4/10	10	C	*bla*_CTX-M-55_	AMP/CTX/TET	IncI1, F18:A-:B1:C4	N	21,977
YZ21HCE10*	feces, F	2021/5/14	12741	B1	*bla*_CTX-M-252_	AMP/CTX/GEN/CHL/FFC/NAL/CIP/CL/SXT	IncI1, IncI2, IncY, F18:A-:B1:C4	*gyrA*(S83L+D87N), *parC* (S80I), *parE* (S458A)	1,663
YZ21HCE12	feces, F	2021/5/14	4373	F	*bla*_CTX-M-64_	AMP/CTX/GEN/STR/TET/CHL/FFC/FOS/SXT	F18:A-:B1:C4, ColpVC	N	160,049
YZ21HCE13	feces, F	2021/5/15	457	F	*bla*_CTX-M-55_	AMP/CTX/GEN/NAL/CIP	F18:A-:B1:C4	*gyrA*(S83L+D87Y), *parC* (S80I+E84G), *parE* (I355T)	382,836
YZ21HCE14*	feces, F	2021/5/15	1049	B1	*bla*_CTX-M-14_	AMP/CTX/GEN/TET/CHL/FFC	IncK, F18:A-:B1:C4	N	96,577
YZ21HCE15	feces, F	2021/5/14	115	E	*bla*_CTX-M-55_	AMP/CTX/STR/TET/CHL/FFC/NAL/SXT	F24:A-:B1, IncI1, IncX1, Col8282, Col156	*gyrA* (S83L), *parE* (I464F)	2,763
YZ21HCE16*	feces, F	2021/5/15	10	C	*bla*_CTX-M-55_	AMP/CTX/TET/CHL/FFC	IncX1, F46:A-:B24	N	46,033
YZ21HCE17	feces, F	2021/5/14	2614	B1	*bla*_CTX-M-65_	AMP/CTX/STR/TET/CHL/FFC/NAL/SXT	IncY, IncQ1	N	3,895
YZ21HCE20	feces, F	2021/5/16	12743	A	*bla*_CTX-M-55_	AMP/CTX/GEN/TET/CHL/FFC/NAL/SXT	IncFIB, IncY	N	38,904
YZ21HCE23*	feces, F	2021/5/16	49	B1	*bla*_CTX-M-14_	AMP/CTX/GEN/TET/CHL/FFC/SXT	F18:A-:B1:C4, IncK	N	65,116
YZ21HCE24*	feces, F	2021/5/16	442	B1	*bla*_CTX-M-55_	AMP/CTX/FOS	F16:A-:B-	N	2,763
YZ21HCE25	feces, F	2021/5/16	12744	B1	*bla*_CTX-M-14_	AMP/CTX/GEN/STR/TET/CHL/FFC/NAL/CIP/FOS/SXT	F55:A-:B6, p0111	*gyrA*(S83L+D87N), *parC* (S80I)	3,320

^a^, * indicates that strain could successfully transfer bla_CTX-M/DHA_ to E. coli C600 by conjugation; Underline indicates nasal and fecal samples obtained from the same individual, YZ21HCE7 and YZ21HEC10, YZ21HCE15 and YZ21HCE18, YZ21HCE17 and YZ21HCE19; Boldface indicates individuals from the same apartment.

^b^, F, female; M, male.

^c^, AMP, ampicillin; CTX, cefotaxime; GEN, gentamicin; STR, streptomycin; TET, tetracycline; CHL, chloramphenicol; FFC, florfenicol; NAL, nalidixic acid; CIP, ciprofloxacin; CL, colistin; FOS, fosfomycin; SXT, sulfamethoxazole/trimethoprim; all strains were susceptible to meropenem and amikacin.

^d^, N, not found.

### Characterization of cefotaxime-resistant *E. coli* isolates

**Figure 1:**
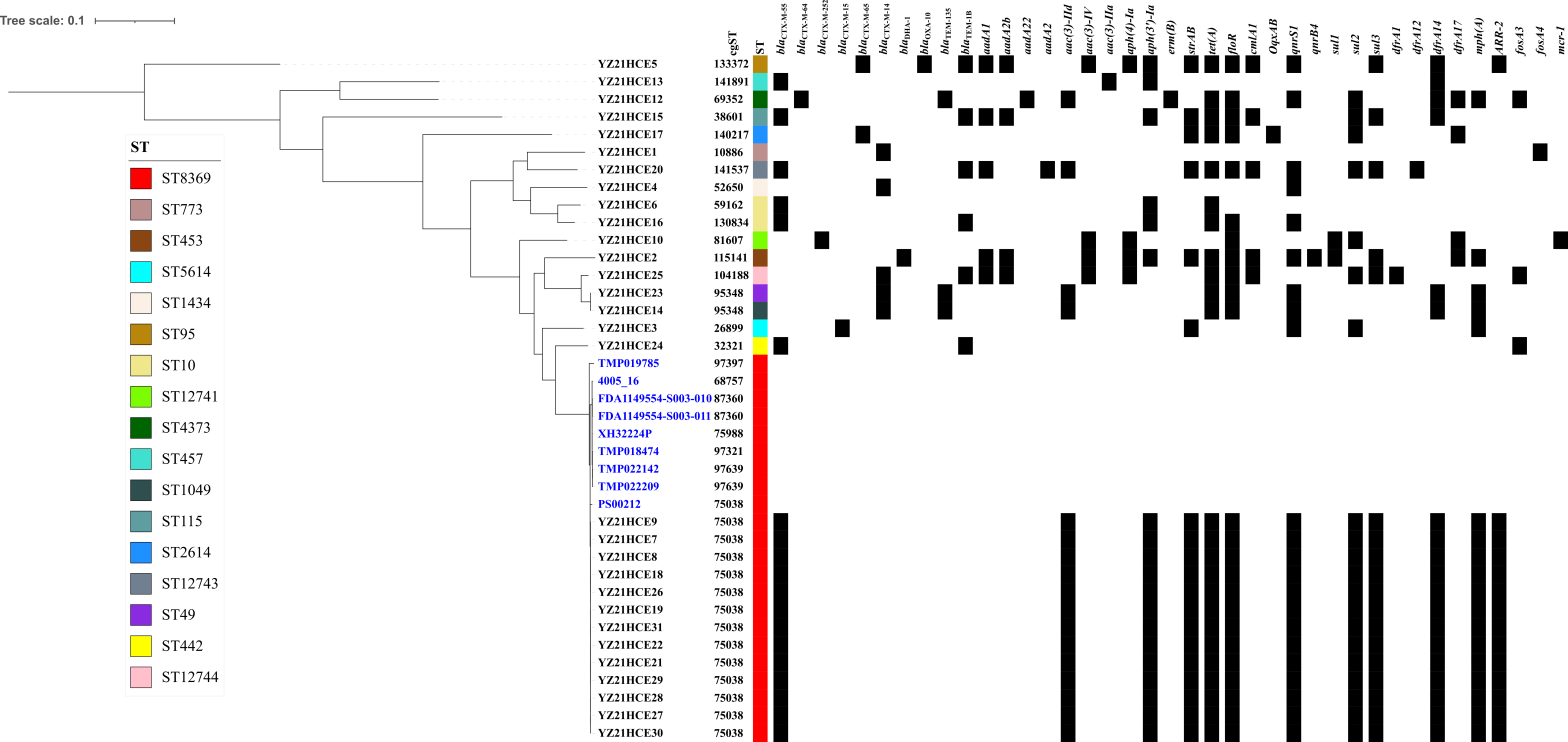
The maximum likelihood tree of cefotaxime-resistant *E. coli* isolates in this study compared with *E. coli* ST8369 isolates from EnteroBase (https://enterobase.warwick.ac.uk/) (in blue) based on cgSNP analysis. Antibiotic resistance genes with >90% sequence homology and coverage are shown.

All ESBL-producing isolates exhibited an MIC of 8 to >128 mg/L to cefotaxime, and also showed resistance to multiple antibiotics, but were susceptible to meropenem and amikacin (**Table 1**). Twenty-two of them successfully transfer cefotaxime resistance to *E. coli* C600 by conjugation (**Table 1**). In addition to *bla*_CTX-M_/*bla*_DHA_, they carried one to 18 resistance genes, such as *bla*_TEM_, *tet*(A), *floR*, *qnrS1*, *fosA3*, and *mcr-1* (**Figure 1**); mutations within *gyrA* (S83L, D87N/Y), *parC* (S80I), or *parE* (I355T, S458A) were observed in six of them (**Table 1**). Phylogenetic group analysis showed that group B1 was predominant (21; 70%), which was frequently associated with commensal or intestinal pathogenic strains (Clermont et al., 2000); followed by group A (3; 10%), group C (2; 6.67%), group F (2; 6.67%) and group E (1; 3.33%) (**Table 1**). Only one CTX-M-65-producing isolate belonged to extraintestinal virulent group B2.

Thirty ESBL-producing isolates were assigned to 14 known STs and three novel STs (ST12741, ST12743 and ST12744) (**Figure 1**). The most prevalent STs among them were ST8369 (n=13). So far, only nine ST8369 *E. coli* isolates were retrieved from EnteroBase (https://enterobase.warwick.ac.uk/) originating from humans, wild animals, and the environment (**Table S1**), and none of them carried resistance genes or mutations associated with quinolone resistance. To reveal the genetic differences between 22 *E. coli* ST8369 isolates, we analyzed their cgMLST profiles (cgSTs) based on 2513 alleles (Zhou et al., 2020). Among the identified seven cgSTs, cgST 75038 (n=14) was the dominant type shared by 13 strains in our study and one isolate PS00212 (**Figure 1**). Thirteen *E. coli* ST8369 strains from nasal (n=12) or fecal (n=1) samples of different individuals in this study shared the same cgST, resistance profiles, resistance genes, and plasmid replicons (**Figure 1** and **Table 1**), indicating that there is a reservoir of this lineage in the community.

### The genetic structures of *bla*_CTX-M_/*bla*_DHA_ in 30 ESBL-producing *E. coli* isolates

**Figure 2:**
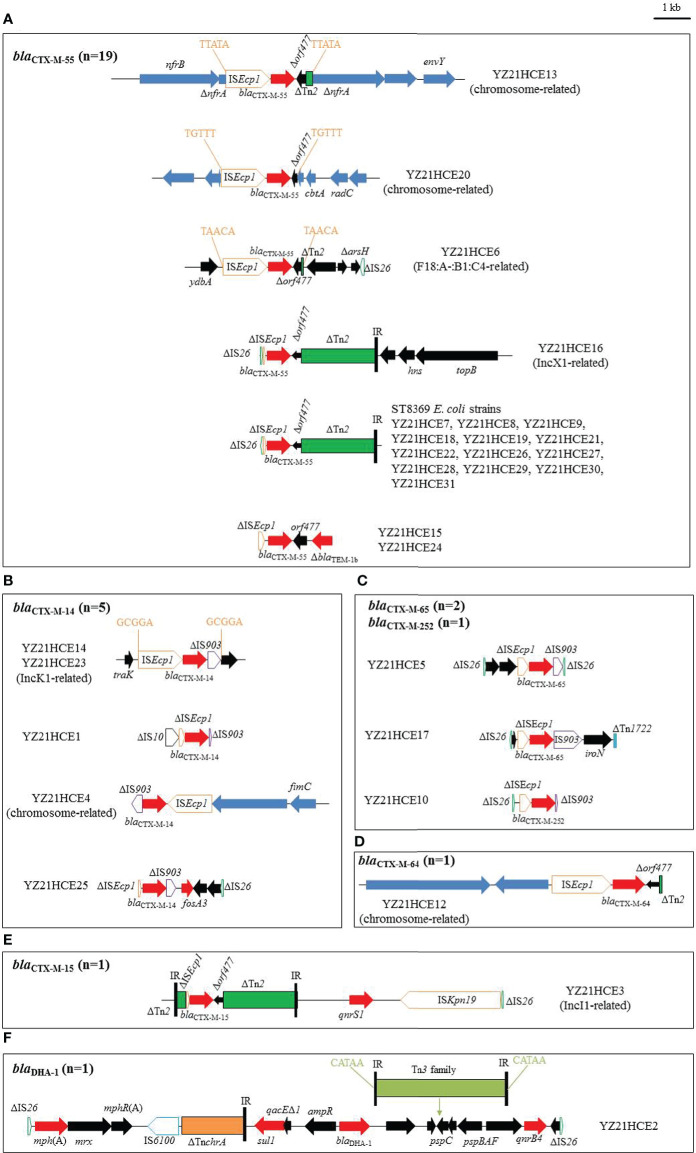
The genetic environments of blaCTX-M/blaDHA in 30 E. coli isolates in this study. **(A)** blaCTX-M-55; **(B)** blaCTX-M-14; **(C)** blaCTX-M-65/-252; **(D)** blaCTX-M-64; **(E)** blaCTX-M-15; **(F)** blaDHA. The extents and directions of antibiotic resistance (red arrows) and other genes (black arrows) are indicated. The blue arrows indicate chromosomal genes. ISs are shown as boxes labeled with their name. Tall bars represent the inverted repeats (IR) of transposon. Δ indicates a truncated gene or mobile element. Arrows and sequences indicate direct repeats.

The lengths of *bla*_CTX-M_/*bla*_DHA_-carrying contigs ranged from 1,663 to 382,836 bp, located on chromosome (n=4) or plasmids (n=5). Twenty-one contigs were short (1,663 to 5,053 bp) due to incomplete assembly and the high number of insertion elements; they did not have replicon genes or plasmid backbone; thus, it is difficult to determine their location (**Table 1**; **Figure 2**).

In YZ21HCE13, the 3,241-bp transposition unit (IS*Ecp1*-*bla*_CTX-M-55_-Δ*orf477*-ΔTn*2*) was inserted into the chromosome and generated 5-bp direct repeats (DRs) (**Figure 2A**). A similar insertion (IS*Ecp1*-*bla*_CTX-M-55_-Δ*orf477*) with a different location had occurred in the chromosome of YZ21HCE20. Similarly, a 3,050-bp segment (IS*Ecp1*-*bla*_CTX-M-55_-Δ*orf477*-ΔTn*2*) with DRs (5’-TAACA-3’) was inserted into the plasmid backbone in YZ21HCE6 (**Figure 2A**), this *bla*_CTX-M-55_-bearing contig (21,977 bp) was identical to those of F18:A-:B1:C4 plasmids, such as pTREC1 (*E. coli*, MN158989) (**Figure S2**). In YZ21HCE16, the *bla*_CTX-M-55_-carrying contig was 46,033 bp in size and similar to IncX1 plasmids such as p40EC-5 (CP070925) and pPK8277-49kb (CP080137) (**Figure S2**). *bla*_CTX-M-55_ was associated with the commonly observed structure ΔIS*26*-ΔIS*Ecp1*-*bla*_CTX-M-55_-Δ*orf477*-ΔTn*2*, and three additional resistance genes *aph(3’)-Ia*, *qnrS1* and *tet*(A) were co-located in this contig (**Figure S2**). The 2,763-bp *bla*_CTX-M-55_ region in isolates YZ21HCE15 and YZ21HCE24 were identical, including the typical transposition unit (ΔIS*Ecp1*-*bla*_CTX-M-55_-*orf477*) and a truncated *bla*_TEM-1b_ downstream, which was commonly observed in many *bla*_CTX-M-55_-carrying plasmids, e.g., pHNMC02 (MG197489). All ST8369 *E. coli* isolates (n=13) had identical 5,053-bp *bla*_CTX-M-55_-positive contigs with structure ΔIS*26*-ΔIS*Ecp1*-*bla*_CTX-M-55_-Δ*orf477*-ΔTn*2*, except strain YZ21HCE29 (one nucleotide change). The complete sequence of YZ21HCE18 as representative ST8369 *E. coli* was obtained (**Table S2**). The *bla*_CTX-M-55_ gene was located on plasmid pYUYZ18-1 with a size of 248,665 bp, belonged to IncHI2/ST3 plasmid with a similar organization to other IncHI2 plasmids. In addition to *bla*_CTX-M-55_, pYUYZ18-1 contained numerous resistance genes, including *aac(3)-IId*, *aph(3’)-Ia*, *strAB*, *tet*(A), *mph*(A), *qnrS1*, *floR*, *sul2*, *sul3*, *dfrA14*, and *arr-2*. Similar pYUYZ18-1 plasmids were also present in other ST8369 *E. coli* strains in this study (**Figure S3**).

Two *bla*_CTX-M-14_-positive contigs from isolates YZ21HCE14 and YZ21HCE23 displayed >99.9% similarity to IncK1 plasmids pD16EC0206-1 (*E. coli*, CP088610) and pJX1-2 (*Klebsiella pneumoniae*, CP064254) (**Figure S2**). The 3,060-bp *bla*_CTX-M-14_ transposition unit (IS*Ecp1*-*bla*_CTX-M-14_-ΔIS*903*) with 5-bp DRs (5’-GCGGA-3’) was inserted downstream of the plasmid conjugal transfer gene *traK* (**Figure 2B**). In isolates YZ21HCE1, YZ21HCE4, and YZ21HCE25, a similar *bla*_CTX-M-14_ transposition unit was observed, differed by deletions involving IS*Ecp1* and/or IS*903*. In YZ21HCE4, the 2,996-bp fragment (IS*Ecp1*-*bla*_CTX-M-14_-ΔIS*903*) was embedded in a 21,609-bp contig associated with the chromosome of *E. coli*. The 1,972-bp *fosA3* segment (*fosA3*-*orf1*-Δ*orf2*-ΔIS*26*) was present downstream of IS*903* in YZ21HCE25 (**Figure 2B**).

As observed in **Figure 2C**, the typical (IS*Ecp1*-*bla*_CTX-M-9G_-IS*903*-*iroN*) transposition unit was found in isolates YZ21HCE5 (*bla*_CTX-M-65_), YZ21HCE17 (*bla*_CTX-M-65_), and YZ21HCE10 (*bla*_CTX-M-252_), although IS*Ecp1* was incomplete. In YZ21HCE17, the *bla*_CTX-M-65_ unit was inserted in an incomplete Tn*1722*. However, IS*903* was truncated by IS*26* at the 3’ end in YZ21HCE5, resulting in the deletions of *iroN* and 701 bp of IS*903*. Similarly, an incomplete IS*903* (80-bp) was also observed in YZ21HCE10. A 1,205-bp structure consisting of two hypothetical proteins plus ΔIS*26* (76-bp) was located upstream of *bla*_CTX-M-65_ transposition unit with a 64-bp spacer in YZ21HCE5. Remnants of this structure were also identified upstream of the *bla*_CTX-M-65_ unit in YZ21HCE17 (214 bp) and YZ21HCE10 (201 bp).

The *bla*_CTX-M-64_ was located on the chromosome of YZ21HCE12. The 160,049-bp *bla*_CTX-M-64_-carrying contig showed highly (>98.0%) similarity to the corresponding region of *E. coli* chromosome such as Z30 (CP066844) and LD67-1 (CP061185). Furthermore, a 3,045-bp region (IS*Ecp1*-*bla*_CTX-M-64_-Δ*orf477*-ΔTn*2*) in YZ21HCE12 was identical to those of chromosome of *E. coli* 3952 (MT773682) and plasmid pM-64-4467-1 (MT773679) from healthy humans in Hangzhou, China (Chen et al., 2021).

The *bla*_CTX-M-15_-positive contig (35,054 bp) in YZ21HCE3 was similar to IncI1 plasmid pBHBSTW-00321_3 (*E. coli*, CP056606) with 86% coverage and 98.36% identity (**Figure S2**). The *bla*_CTX-M-15_ gene was associated with genetic content ΔTn*2*-ΔIS*Ecp1*-*bla*_CTX-M-15_-Δ*orf477*-ΔTn*2*, seen in several plasmids, such as p5908-2 (*Shigella flexneri*, CP045523) and p92 (*E. coli*, CP041521). The *bla*_CTX-M-15_ resistance module was followed by a 5,752-bp structure *qnrS1*-IS*Kpn19*-ΔIS*26* (**Figure 2E**).

The *bla*_DHA_-carrying contig (18,905 bp) of YZ21HCE2 was identical to the corresponding regions of multiple plasmids such as pM2901 (*Shigella sonnei*, CP061363), except for the insertion of one Tn*3* family transposon within *pspC* flanked by 5-bp DRs in YZ21HCE2. The core structure *sul1*-*qacEΔ1*-*ampR*-*bla*_DHA-1_-*pspDCBAF*-*qnrB4* is commonly observed in numerous plasmids from various species (e.g., *Salmonella*, *Klebsiella pneumoniae*, *Citrobacter freundii*), highlighting the co-transfer ability of *bla*_DHA-1_ and *qnrB4*.

## Discussion

The *bla*_CTX-M_ gene has been globally disseminated in different sources, with *bla*_CTX-M-14_ and *bla*_CTX-M-15_ being dominant (Bevan et al., 2017). In this study, *bla*_CTX-M-55_ is the most predominant genotype in healthy individuals, which agrees with the increasing prevalence of *bla*_CTX-M-55_ in both animals and patients in China (Bevan et al., 2017). As a variant of *bla*_CTX-M-15_, *bla*_CTX-M-55_ was first reported in clinical *E. coli* and *K. pneumoniae* isolates in Thailand in 2007 (Kiratisin et al., 2007). Recently, *bla*_CTX-M-55_ has become the predominant CTX-M genotype in *E. coli* and *Salmonella* from food animals, food products, and patients in China (Rao et al., 2014; Zhang et al., 2014; Fu et al., 2020; Huang et al., 2020; Liu et al., 2022; Zeng et al., 2022). One novel *bla*_CTX-M_ variant *bla*_CTX-M-252_ was identified in this study. CTX-M-252 and CTX-M-65 differ by a single amino acid and share a similar genetic structure, suggesting a common lineage. Further investigation of *bla*_CTX-M-252_ is needed.

In communities, faecal carriage rates of *bla*_CTX-M_ are increasing, particularly in developing countries (Woerther et al., 2013; Bevan et al., 2017). However, investigation of *bla*_CTX-M_ in nasal samples of healthy humans is rare. Previously, one (1/77, 1.30%) CTX-M-producing *E. coli* isolate was obtained from the nasal sample of a healthy human working at a pig abattoir in Cameroon (Founou et al., 2018). A high nasal carriage rate (32.43%) of *bla*_CTX-M_ was observed in this study, and nasal colonization of ST8369 *E. coli* producing CTX-M-55 among healthy persons occurred in one community in Yangzhou, China. It suggests that nasal carriage of *bla*_CTX-M_ is possibly common in humans. However, the small number of samples and communities is a limitation of this study. The acquisition of *bla*_CTX-M-55_ and other resistance genes by ST8369 is mediated by the horizontal transfer of IncHI2 plasmid, followed by clonal dissemination. *E. coli* ST8369 is rarely described worldwide and may represent an emerging clone in humans, animals, and the environment. Nasal colonization of *bla*_CTX-M-55_-carrying ST8369 *E. coli* suggests a potential risk of antimicrobial resistance dissemination between humans by the spread of clonal lineages in the small-scale community through close contact or environment *via* aerosols or dust. Therefore, the clinical importance of nasal carriage of CTX-M-producing *E. coli* might be underestimated. Although horizontal transfer is the main reason for *bla*_CTX-M_ dissemination, clonal spread of *bla*_CTX-M_-harbouring strains, such as *E. coli* ST8369 in this study, CTX-M-15-producing *E. coli* ST949 in water surfaces, *E. coli* ST2179 encoding CTX-M-65 in retail meat, and *bla*_CTX-M-55_-carrying *Salmonella* Typhimurium ST34 in patients (Bevan et al., 2017; Falgenhauer et al., 2021; Leão et al., 2021; Zeng et al., 2022) is another important route for *bla*_CTX-M_ transmission.

Horizontal transfer mediated by plasmids and mobile elements is responsible for the global spread of *bla*_CTX-M_ (Bevan et al., 2017; Partridge et al., 2018). For example, IncI, IncFII, and IncHI2 plasmids facilitate the horizontal transmission of *bla*_CTX-M_ in *E. coli* and *Salmonella* from various sources (Chen et al., 2021; Guo and Zhao, 2021; Yang et al., 2014; Nadimpalli et al., 2019; Zhang et al., 2021; Zeng et al., 2021). In this study, various plasmids such as IncHI2, IncK1, IncX1, and IncI1 were associated with *bla*_CTX-M_. Although we were not able to determine the location of *bla*_CTX-M_ in some *E. coli* isolates in this study due to incomplete assembly, sequence analysis indicates that IS*Ecp1* plays an important role in *bla*_CTX-M_ dissemination among *E. coli* isolates and facilitates the horizontal transfer of *bla*_CTX-M_ from plasmids to chromosomes in distinct integration sites. The chromosomal integration of *bla*_CTX-M_ is increasingly reported in *E. coli*, *K. pneumoniae*, *Salmonella*, *Proteus mirabilis* and some other species of Enterobacteriaceae with the help of mobile elements (Huang et al., 2017; He et al., 2017; Zeng et al., 2022; Yoon et al., 2022). Chromosomal integration of *bla*_CTX-M_ seems to be an adaptive evolution in response to antimicrobial pressure (Yoon et al., 2022).

In conclusion, we report nasal colonization of CTX-M-55-producing *E. coli* ST8369 associated with IncHI2 plasmid in healthy individuals in one community from Yangzhou, China. Therefore, continued surveillance of nasal carriage of *bla*_CTX-M_ in communities is warranted.

### Accession Numbers

The sequences have been deposited in the GenBank under accession number: PRJNA819533.

## Data availability statement

The datasets presented in this study can be found in online repositories. The names of the repository/repositories and accession number(s) can be found in the article/**Supplementary Material**.

## Ethics statement

The studies involving human participants were reviewed and approved by Yangzhou University. The participants provided their written informed consent to participate in this study.

## Author contributions

JW and Q-CL conceived and designed the experiments, Z-YW, YJ, Y-QS, H-FL, and M-JL carried out the experiments. Z-YW and JW analyzed the data and wrote the manuscript. Q-CL and XJ revised the manuscript. All authors contributed to the article and approved the submitted version.

## Funding

This study was supported by the National Natural Science Foundation of China (no. 31902319), the fifth phase of the “333 project” scientific research project in Jiangsu Province (no. BRA2020002), and Postgraduate Research &Practice Innovation Program of Jiangsu Province (Yangzhou University) (no. KYCX22_3536).

## Conflict of interest

The authors declare that the research was conducted in the absence of any commercial or financial relationships that could be construed as a potential conflict of interest.

## Publisher’s note

All claims expressed in this article are solely those of the authors and do not necessarily represent those of their affiliated organizations, or those of the publisher, the editors and the reviewers. Any product that may be evaluated in this article, or claim that may be made by its manufacturer, is not guaranteed or endorsed by the publisher.
