# Supplementary figures and images for: Nasal carriage of CTX-M-55-producing Escherichia coli ST8369 in a healthy cohort in the city of Yangzhou, China
Source: Front Cell Infect Microbiol. 2022 Aug 3;12:970940. doi: 10.3389/fcimb.2022.970940 (PMC9382594; doi:10.3389/fcimb.2022.970940)

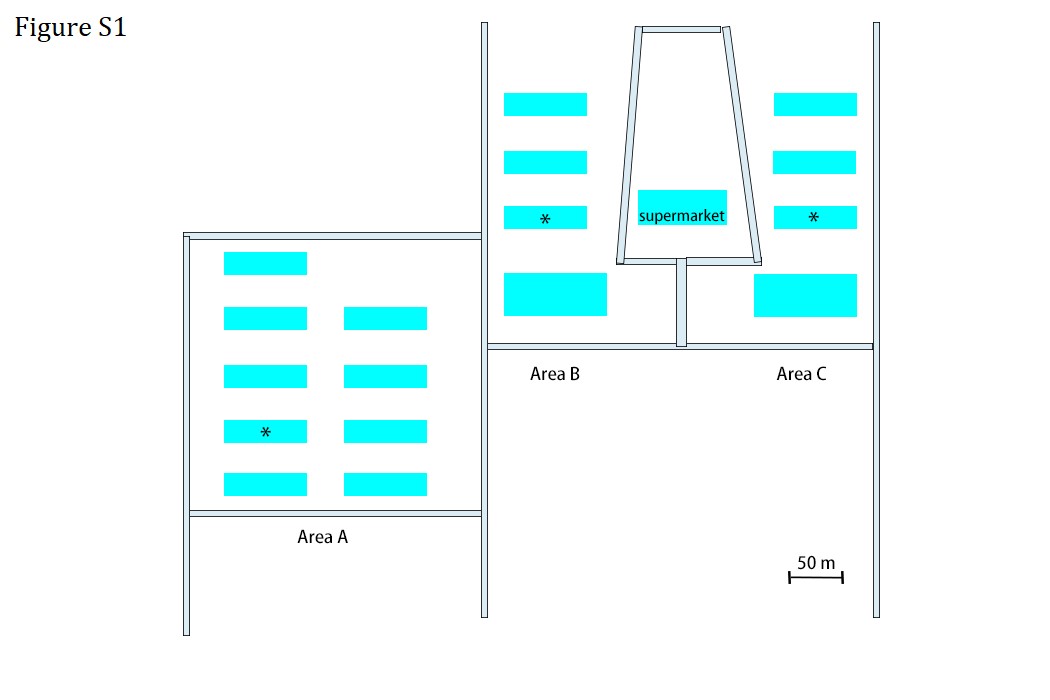

Supplement: Supplementary file 1 [file Image_1.jpeg]

Figure S2

A

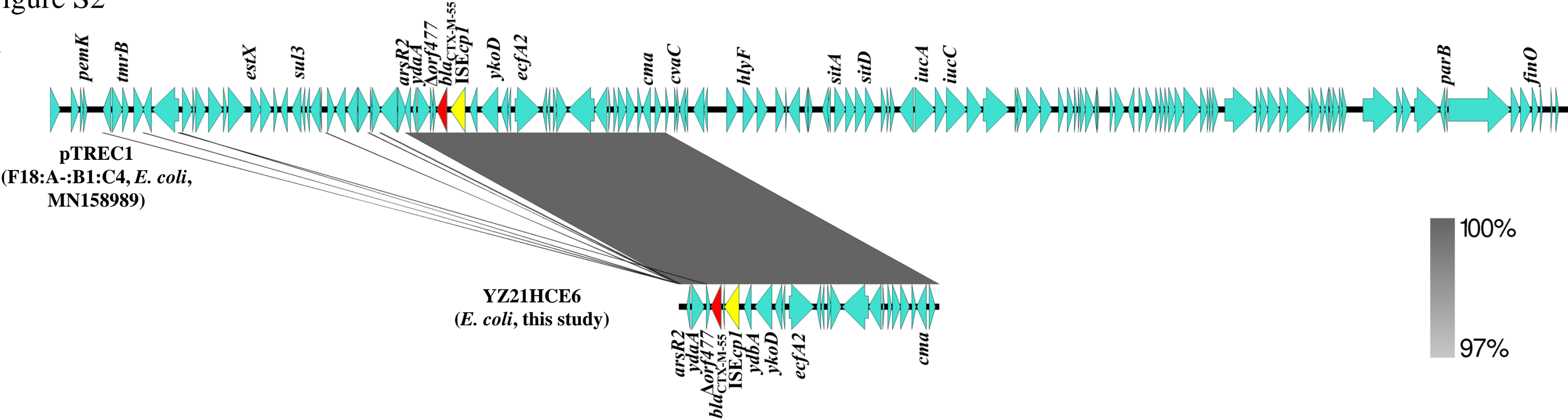

B

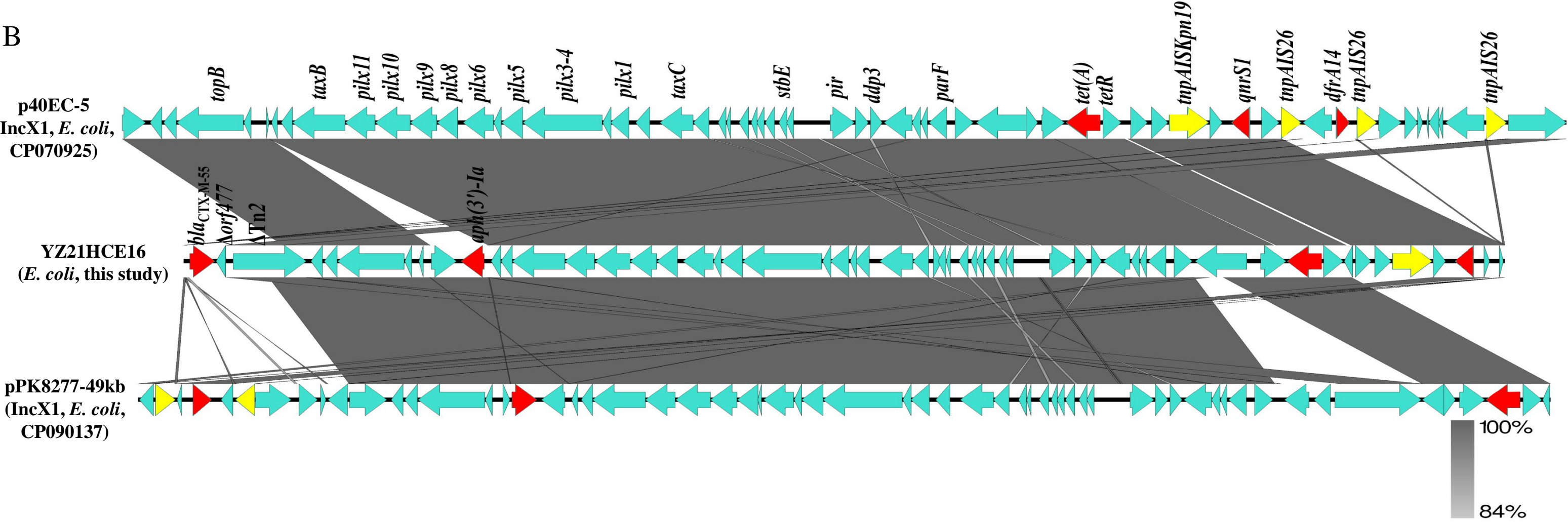

C

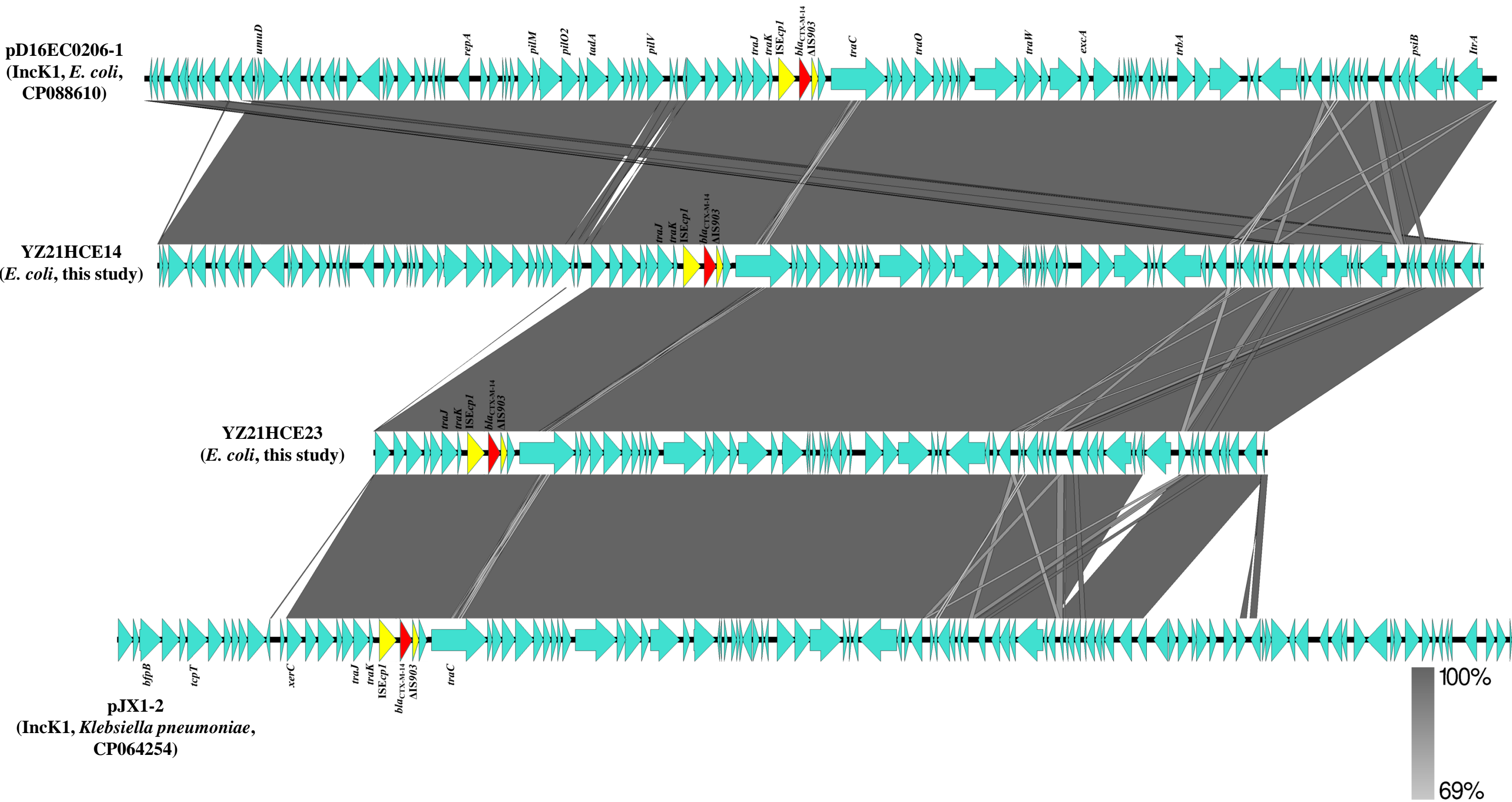

D

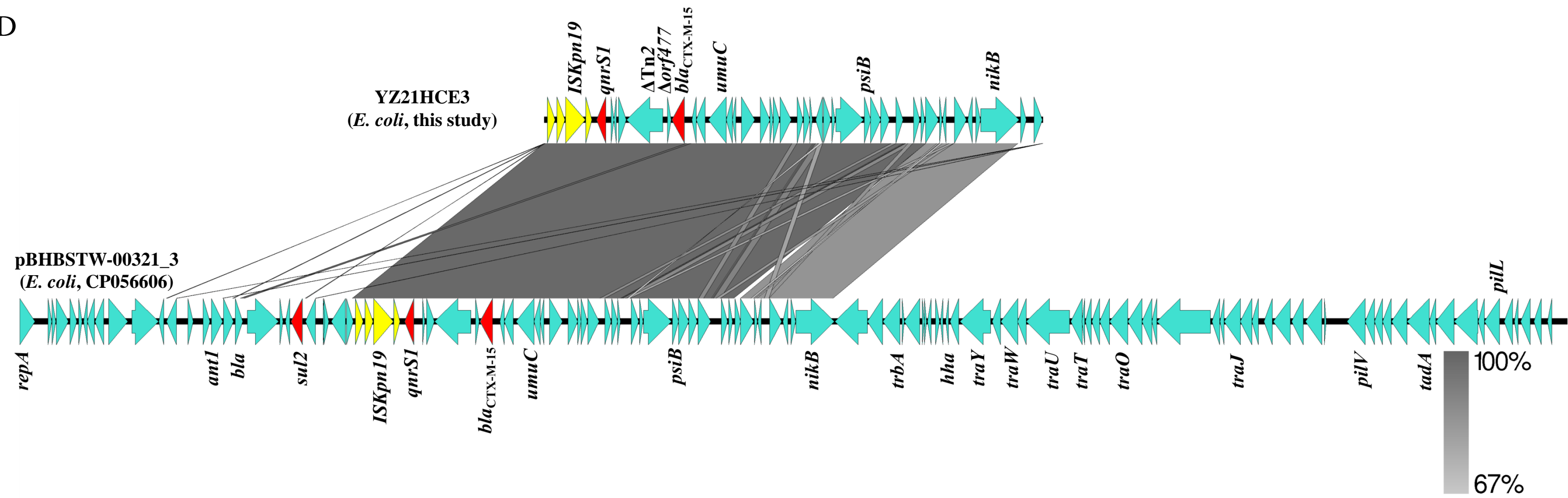

Supplement: Supplementary file 3 [file DataSheet_2.pdf]

Figure S3

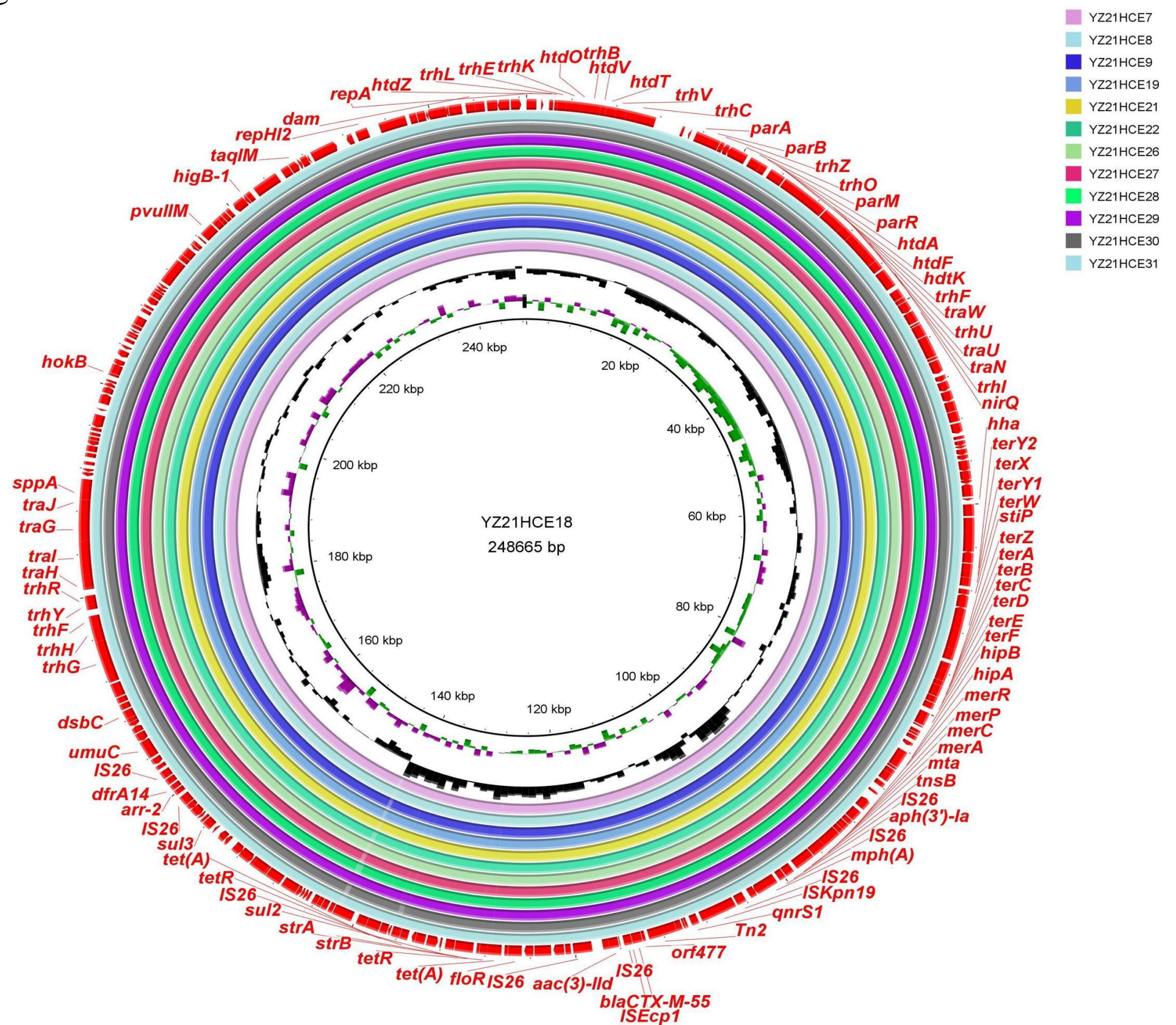

Supplement: Supplementary file 4 [file DataSheet_3.pdf]
