# Supplementary material for: Nasal carriage of CTX-M-55-producing Escherichia coli ST8369 in a healthy cohort in the city of Yangzhou, China
Source: Front Cell Infect Microbiol. 2022 Aug 3;12:970940. doi: 10.3389/fcimb.2022.970940 (PMC9382594; doi:10.3389/fcimb.2022.970940)
Supplement: Supplementary file 2 [file DataSheet_1.pdf]

Table S1 ST8369 *Escherichia coli* isolates recorded in EnteroBase (https://enterobase.warwick.ac.uk).

Strains	Source	Collection time	Country	Accession no.
4005/16	human	2016	Germany	NA
PS00212	NA	2018	NA	NA
XH32224P	NA	2013	NA	NA
TMP022209	wild animal	3/2016	Kenya	ERR3330420
TMP022142	wild animal	3/2016	Kenya	ERR3330419
TMP019785	human	11/2015	Kenya	ERR3330221
TMP018474	wild animal	2015	Kenya	ERR3330420
FDA114955-S003-010	environment	10/2020	United State	SRR1292713
FDA114955-S003-011	environment	10/2020	United State	SRR1292713

NA: not available.

Table S2 Whole genome of ST8369 *Escherichia coli* strain YZ21HCE18 in this study.

	Size (bp)	Resistance genes	Plasmid replicon
YZ21HCE18			
chromosome	4,728,170	*mdf*(A)	
pYUYZH18-1	248,665	$bla_{CTX-M-55}$, *aac(3)-IId*, *aph(3')-Ia*, *strAB*, *tet*(A), *qnrS1*, *floR*, *mph*(A), *sul2*, *sul3*, *dfrA14*, *arr-2*	IncHI2
pYUYZH18-2	90,135	*strAB*, *tet*(A), *qnrS1*, *sul2*, *dfrA14*	F102:A17:B-

Figure S1. The locations of buildings sampled in one community. * indicates the building we sampled.

Figure S2. Linear sequence comparison of bla_{CTX-M}-carrying contig (plasmid-related) with other similar plasmids using Easyfig.

Figure S3. Sequence comparison of $bla_{CTX-M-55}$-carrying plasmids from ST8369 *E. coli* strains in this study with plasmid pYUYZH18-1 from ST8369 *E. coli* isolate YZ21HCE18 using BRIG. The reference sequence pYUYZH18-1 is indicated in red in the outer circle.
